# “Gate-keeper” Residues and Active-Site Rearrangements in DNA Polymerase μ Help Discriminate Non-cognate Nucleotides

**DOI:** 10.1371/journal.pcbi.1003074

**Published:** 2013-05-23

**Authors:** Yunlang Li, Tamar Schlick

**Affiliations:** Department of Chemistry and Courant Institute of Mathematical Sciences, New York University, New York, New York, United States of America; Michigan State University, United States of America

## Abstract

Incorporating the cognate instead of non-cognate substrates is crucial for DNA polymerase function. Here we analyze molecular dynamics simulations of DNA polymerase μ (pol μ) bound to different non-cognate incoming nucleotides including A:dCTP, A:dGTP, A(syn):dGTP, A:dATP, A(syn):dATP, T:dCTP, and T:dGTP to study the structure-function relationships involved with aberrant base pairs in the conformational pathway; while a pol μ complex with the A:dTTP base pair is available, no solved non-cognate structures are available. We observe distinct differences of the non-cognate systems compared to the cognate system. Specifically, the motions of active-site residue His329 and Asp330 distort the active site, and Trp436, Gln440, Glu443 and Arg444 tend to tighten the nucleotide-binding pocket when non-cognate nucleotides are bound; the latter effect may further lead to an altered electrostatic potential within the active site. That most of these “gate-keeper” residues are located farther apart from the upstream primer in pol μ, compared to other X family members, also suggests an interesting relation to pol μ's ability to incorporate nucleotides when the upstream primer is not paired. By examining the correlated motions within pol μ complexes, we also observe different patterns of correlations between non-cognate systems and the cognate system, especially decreased interactions between the incoming nucleotides and the nucleotide-binding pocket. Altered correlated motions in non-cognate systems agree with our recently proposed hybrid conformational selection/induced-fit models. Taken together, our studies propose the following order for difficulty of non-cognate system insertions by pol μ: T:dGTP<A(syn):dATP<T:dCTP<A:dGTP<A(syn):dGTP<A:dCTP<A:dATP. This sequence agrees with available kinetic data for non-cognate nucleotide insertions, with the exception of A:dGTP, which may be more sensitive to the template sequence. The structures and conformational aspects predicted here are experimentally testable.

## Author Summary

DNA polymerase μ (pol μ) is an enzyme that participates in DNA repair and thus has a central role in maintaining the integrity of genetic information. To efficiently repair the DNA, discriminating the cognate instead of non-cognate nucleotides (“fidelity-checking”) is required. Here we analyze molecular dynamics simulations of pol μ bound to different non-cognate nucleotides to study the structure-function relationships involved in the fidelity-checking mechanism of pol μ on the atomic level. Our results suggest that His329, Asp330, Trp436, Gln440, Glu443, and Arg444 are of great importance for pol μ's fidelity-checking mechanism. We also observe altered patterns of correlated motions within pol μ complex when non-cognate instead of cognate nucleotides are bound, which agrees with our recently proposed hybrid conformational selection/induced-fit models. Taken together, our studies help interpret the available kinetic data of various non-cognate nucleotide insertions by pol μ. We also suggest experimentally testable predictions; for example, a point mutation like E443M may reduce the ability of pol μ to insert the cognate more than of non-cognate nucleotides. Our studies suggest an interesting relation to pol μ's unique ability to incorporate nucleotides when the upstream primer is not paired.

## Introduction

The integrity of genetic information depends largely on DNA polymerases that are central to DNA replication, damage repair, and recombination. DNA polymerase errors are associated with numerous diseases, including various cancers and neurological conditions [1]–[13]. One of the most basic types of errors that DNA polymerases generate is base substitution error, which means that DNA polymerase inserts an non-cognate (“non-cognate”) nucleotide opposite the DNA template base to form a nonstandard base pair (i.e., A:dATP base pair, instead of A:dTTP base pair). Although DNA polymerases conduct similar nucleotidyl transfer reaction and share a similar structure - palm, thumb and fingers subdomains [14], they can exhibit varying levels of accuracy (“fidelity”) in inserting nucleotides [15].

DNA polymerase μ (pol μ) of the X-family, like the other X-family members, participates mainly in DNA repair rather than replication [16]. Like two other X-family members polymerase β (pol β) and polymerase λ (pol λ), pol μ can bind to DNA and fill single-strand DNA gaps in a template-dependent manner with moderate fidelity (10^−4^–10^−5^) [17]–[19]. Furthermore, like another X-family member terminal deoxynucleotidyl transferase (Tdt), pol μ can also perform nucleotide insertion in a template-independent manner [20], [21]. In addition, pol μ can direct template-based DNA synthesis without requiring all upstream primer bases to be paired [17], [22]. The unique substrate flexibility of pol μ may signal a unique role in the nonhomologous DNA end joining (NHEJ) process for double-strand breaks in DNA and V(D)J recombination [22]–[28].

**Figure 1:**
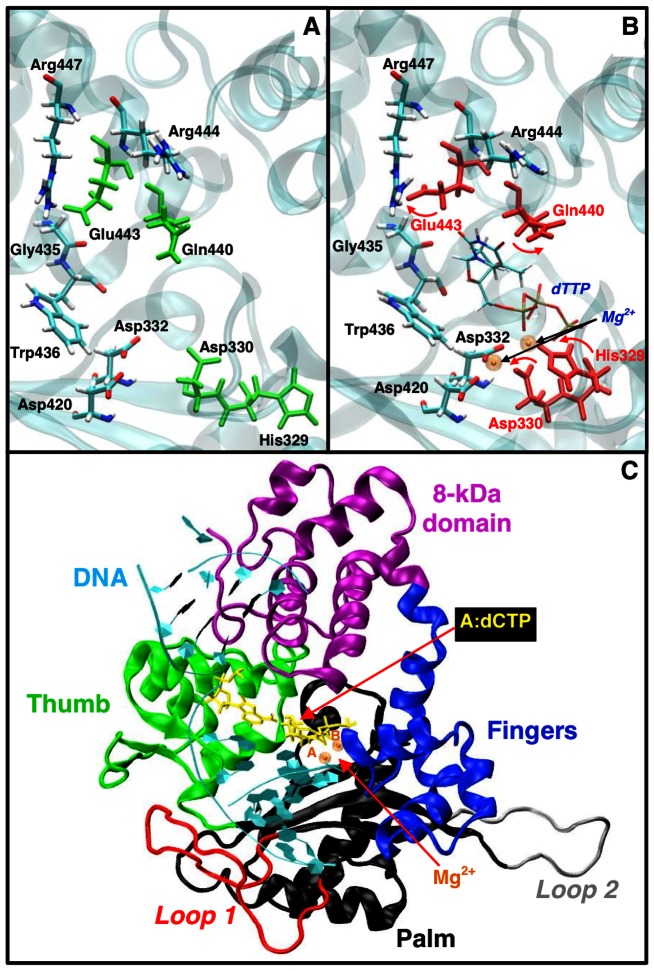
Structure of pol μ without and with cognate/non-cognate substrates. (A) structure without incoming nucleotide (inactive); (B) structure with cognate incoming nucleotide (active); (C) our starting model of the pol μ/DNA/dCTP non-cognate system. Mg^2+^(A), catalytic ion; Mg^2+^(B), nucleotide-binding ion. Key residues with conformational changes are marked as green in (A) and red in (B), respectively.

Structural and computational studies have uncovered important differences and similarities regarding how pol μ incorporate a cognate nucleotide into single-nucleotide gapped DNA, compared to other X-family members [29], [30]. For pol β, upon binding the cognate incoming nucleotide, the enzyme undergoes a large-scale protein motion in the thumb subdomain from open (inactive) to closed (active) conformation [31]–[33]. Such open-to-closed protein motion is also observed in pol X, another X-family polymerase [34]. Pol λ lacks such large-scale protein transitions; instead, a large shift of the DNA template from the inactive to the active state is indicated by both crystal structures [35] and simulations [36]. The large-scale protein motion in pol β and pol X or DNA motion in pol λ is crucial for the polymerization activity [31], [37], [38]. In pol μ, studies have suggested the lack of significant DNA or protein motion before chemistry [29]. Pol μ shares with pol β and pol λ the notion that subtle active-site protein residue motions help organize the conformation of the active site and prepare for the following chemical step [39], but the specific residues are different [29], [33], [36]. In pol μ, His329 and Asp330 assemble pol μ's active site, and Gln440 and Glu443 help accommodate the incoming nucleotide. See Fig. 1(a) and (b) for key residues and their motion in pol μ's cognate system.

In prior mismatch studies on various X-family DNA polymerases such as pol β [40]–[45], pol X [46], and pol λ [47], reduced large-scale protein (pol β and pol X) or DNA motions (pol λ) were observed, related to the inactivity of non-cognate systems. Varying amounts of active-site distortions are observed. Distortions of the active site are caused by the conformational changes of several key residues (“gate-keepers”) [40], [47]. For example, in pol β, structural and dynamics analyses revealed different behavior of Arg258, Asp192, and Phe272 in non-cognate systems [40]–[43]. These residues distort the active site, with the degree of active-site distortions system-dependent and in accord with the sequence of kinetic data for non-cognate base pair incorporations. The different conformational behavior between the cognate and non-cognate systems before and/or after chemistry are also observed and are related to fidelity for DNA polymerases in other families [48]–[51].

From prior results, we further demonstrated that characteristic motions recur within various 2′-deoxyribonucleoside 5′-triphosphate (dNTP) contexts. Specifically, correlated protein and dNTP motions occur within cognate dNTP complexes and are altered within non-cognate dNTP complexes. We therefore proposed a hybrid conformational selection/induced-fit model for DNA polymerases [52]. In this model, the cognate dNTP selectively binds to a near-active conformation from an ensemble of possible polymerase/DNA conformations, and then the bound dNTP induces small adjustments within the active site, driving the complex to a fully-active state ready for catalysis. Non-cognate dNTPs that are relatively efficiently handled by the polymerase would also selectively bind to a near-active conformation, but the active-site changes induced by the non-cognate dNTP binding would differ from those by cognate dNTP binding. For non-cognate dNTPs that are relatively poorly inserted by the polymerase, dNTP may bind to a variable inactive conformation. The resulting incomplete organization of the active site would reduce the efficiency for inserting an non-cognate dNTP. This proposed broader view better reflects both the intrinsic motions of polymerases and the highly specific nature of polymerase/ligand interactions, and has gained further support from additional computations [53]–[57] (Arora, Zahran, and Schlick, in preparation).

Several key experimental studies of pol μ's fidelity exist [17], [19], but no structure of an non-cognate incoming nucleotide bound to pol μ has been reported. Modeling and all-atom dynamics simulations can help study the structural and dynamic properties of non-cognate pol μ systems, which in turn can be related to specific functions of pol μ. Needless to say, all dynamics simulation data are subject to the approximations and limitations of an empirical force field, limited sampling, and large computational requirements [58]. Yet, modeling and simulation have demonstrated many successes in biomolecular structure and function problems, and can be valuable especially when few experimental data are available [59].

In this study, we investigate dynamics of pol μ bound to various mismatches (A:dCTP, A:dATP, A:dGTP, T:dCTP, and T:dGTP) to determine the factors that contribute to insertion differences of pol μ during its conformational pathway before chemistry. We also analyze simulations of the bulky purine-purine mismatches with the template base in both the *anti* and *syn* orientations to determine whether particular base pair geometry might facilitate mismatch incorporation. We find that His329 and Asp330 near the active site help discriminate cognate from non-cognate incoming nucleotides. In addition, we suggest that Trp436, Gln440, Glu443, and Arg444 play the role of “gate-keepers” in pol μ by tightening (deactivating) the nucleotide-binding pocket when non-cognate nucleotides are bound. Compared to pol β and pol λ, most of these residues are much farther from the upstream primer in pol μ. A comparison of the correlated motions in cognate and non-cognate pol μ systems indicates decreased interactions in non-cognate systems, especially those between the incoming nucleotides and the nucleotide-binding pocket, and suggests that pol μ also fits into the hybrid conformational selection/induced-fit model. As in pol β and pol λ, the degree of active-site distortion in pol μ mirrors trends in kinetic data, except for A:dGTP, which is more disordered and sequence-context dependent as indicated by kinetic data. Though the chemical step can also impact the fidelity of pol μ, the conformational pathway is a pre-requisite for chemistry [39]. Indeed, in non-cognate systems, the conformational pathway produces a deformed active site that is farther from the chemistry competent state. Thus, even if the chemical step is hindered in non-cognate systems, it is the distorted conformational pathway that leads to initial hindrance in the chemical pathway. Finally, we suggest that the ability of pol μ to incorporate nucleotides when the upstream primer is not paired may arise in part from the fact that most “gate-keeper” residues in pol μ are much farther from the upstream primer, compared to pol β and pol λ; thus, pol μ may be less sensitive to changes around the upstream primer.

## Materials and Methods

### Initial Models

Seven pol μ non-cognate models were prepared on the basis of the X-ray crystal murine pol μ cognate ternary complex (PDB entry 2IHM) [30]. In the crystal structure, two loops in the palm (Loop1, His366-Arg389; Loop2, Pro397-Cys411) are partially missing. Missing protein residues His366-Val386 and Ala403-Ala405 were inserted with the InsightII package (Accelrys Inc., San Diego, CA). A hydroxyl group was added to the 3′ carbon of the 2′,3′-dideoxythymidine 5′-triphosphate (ddTTP) sugar moiety to form 2′-deoxythymidine 5′-triphosphate (dTTP). All other missing atoms from the crystal structure were similarly added. The Na^+^ occupying the catalytic ion site in the crystal structure was modified to Mg^2+^. In our previous study on pol μ cognate system [29], we observed that different protonation states of His329 do not affect the geometry of active site or the conformation of key residues significantly. Therefore, in this study, we only modeled His329 in its default protonation state (Nδ).

In each model, the A:dTTP nascent base pair was replaced with a different non-cognate base pair; namely, A:dCTP, A:dATP, A:dGTP, T:dCTP, or T:dGTP (the template base's symbol is written first, followed by the incoming nucleotide's symbol). Purine bases can assume both *anti* and *syn* orientations. Because a crystal structure of pol β with a template base in *syn* conformation has been reported [60], we modeled the template adenine in the A:dATP and A:dGTP systems in both orientations. The protein residues and other DNA base sequences remain unchanged. We also built a cognate T:dATP system to discern similarities of cognate base pairs.

All models were solvated with explicit TIP3 water model in a water box using the VMD program [61]. The smallest image distance between the solute and the faces of the periodic cubic cell was 7 Å. Besides the water molecules in the crystal structure, 13,625 water molecules were added into each model using VMD program. The total number of water molecules is 13,716. To obtain a neutral system at an ionic strength of 150 mM, 46 Na^+^ and 28 Cl^−^ ions were added to each system. All of the Na^+^ and Cl^−^ ions were placed at least 8 Å away from both protein and DNA atoms and from each other.

All initial models contained approximately 47,621 atoms, 91 crystallographically resolved water molecules from the ternary complex, 13,625 bulk water molecules, 2 Mg^2+^ ions, incoming nucleotide dNTP, and 46 Na^+^ and 28 Cl^−^ counter-ions.

### Minimization, Equilibration, and Dynamics Protocol

Initial energy minimizations and equilibration simulations were performed using the CHARMM program (version c35b2) [62] with the CHARMM all-atom force field including the cross term energy correction map (CMAP) specification for proteins [63]–[65]. The system was minimized with fixed positions for all heavy atoms of protein or nucleotides, using SD for 10,000 steps followed by ABNR for 20,000 steps. Then the atoms of added residues (His366-Val386 and Ala403-Ala405) and non-cognate nucleotide base-pair were released. Another cycle of minimization was performed for 10,000 steps using SD followed by 20,000 steps of ABNR. The equilibration process was started with a 100 ps simulation at 300 K using single-time step Langevin dynamics, while keeping all the heavy atoms of protein or nucleotides fixed. The SHAKE algorithm [66] was employed to constrain the bonds involving hydrogen atoms. This was followed by unconstrained minimization consisting of 10,000 steps of SD and 20,000 steps of ABNR.

The missing loop construction was performed using the program NAMD [67] with the CHARMM force field. All protein or DNA atoms were fixed, except those from the added residues (His366-Val386 and Ala403-Ala405) and the non-cognate base-pair in order to relax the added loop, the non-cognate base-pair, and the water around our complexes. Each system was equilibrated for 1 ns at constant pressure and temperature. Pressure was maintained at 1 atm using the Langevin piston method [68] with a piston period of 100 fs, a damping time constant of 50 fs and a piston temperature of 300 K; the temperature was maintained at 300 K using weakly coupled Langevin dynamics of non-hydrogen atoms with a damping coefficient of 10 ps^−1^. Bonds to all hydrogen atoms were kept rigid using SHAKE, permitting a time step of 2 fs. The system was simulated in periodic boundary conditions with full electrostatics computed using the PME method [69] with grid spacing on the order of 1 Å or less. Short-range non-bonded terms were evaluated at every step using a 12 Å cutoff for van der Waals interactions and a smooth switching function. Molecular dynamics at a constant temperature and volume for 4 ns were followed, using the same constraints as above. The final dimension of each system is approximately 78.95 Å × 74.61 Å × 79.91 Å. The model of the A:dCTP system is shown in Fig. 1(c) as an example.

In prior study, we found that the conformation of the added Loop1 does not affect the behavior of pol μ system significantly [29]. In addition, Loop1 is far away from the active-site region we are interested in. Therefore, we only modeled one conformation of Loop1 for all systems.

Production dynamics were also performed using the NAMD program with the CHARMM force field. In all trajectories, all heavy atoms were free to move. Each simulation was run for 120 ns. Molecular dynamics simulations using the NAMD package were run on the IBM Blue Gene/L at Rensselaer Polytechnic Institute and the Dell computer cluster at New York University.

## Results/Discussion

### Lack of Large-scale DNA and Protein Motions

No substantial protein subdomain or DNA motions were captured during all our non-cognate simulations (Fig. S1). This agrees with our prior suggestion that unlike pol β or pol λ, an open-to-closed transition characterized by large-scale protein or DNA motions may not exist in pol μ [29]. Due to the larger size of dGTP and dATP than that of the cognate dTTP, the template base A5 at the gap pairing with dNTP shifts from its original position significantly (at 95% confidence level, Fig. S1(b) and Fig. S2) in A:dGTP and A:dATP non-cognate systems, to better accommodate the incoming nucleotide. In the A:dCTP system, dCTP is relatively smaller, therefore dCTP can be accommodated without the shift of A5. However, the shift of the single base A5 does not incur wide range movements in DNA backbones of pol μ complexes. This agrees with our prior work that pol μ binds to the DNA more tightly than pol λ [29].

### Active-Site Distortions

**Figure 2:**
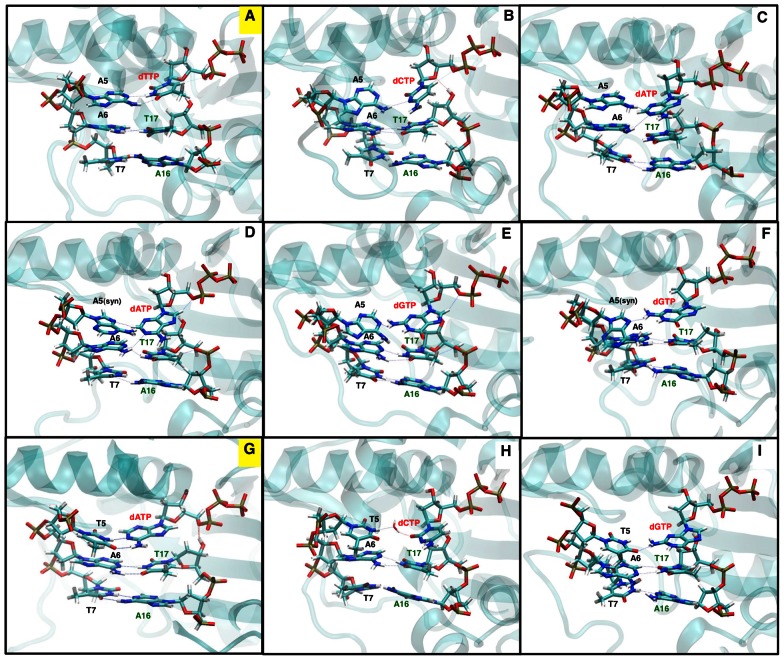
Conformational comparison of active-site base-pairs and their two neighboring base-pairs. (A) A:dTTP cognate system; (B) A:dCTP non-cognate system; (C) A:dATP non-cognate system; (D) A(syn):dATP non-cognate system; (E) A:dGTP non-cognate system; (F) A(syn):dGTP non-cognate system; (G) T:dATP cognate system; (H) T:dCTP non-cognate system; (I) T:dGTP non-cognate system. The two cognate systems (A and G) are labeled in yellow. Dashed lines indicate hydrogen bonds.

Active sites in the non-cognate systems are significantly distorted compared to those in the cognate systems because the Watson-Crick base-pair between the incoming nucleotide and its corresponding template base no longer exist (Fig. 2). New hydrogen bonds between those two bases form (directly, or through a water molecule in T:dCTP system). However, these new hydrogen bonds are less stable than those in the Watson-Crick base pair. In addition, the steric hindrance between the two large purine bases in purine:purine non-cognate systems like A:dATP and A:dGTP further destabilize their interactions. Thus, the nucleotide fluctuates substantially within the active site, indicating a lower active-site conformational stability. In the A:dATP and A(syn):dATP systems, the non-cognate dATP interacts with both A5 and A6 in the template, without breaking the hydrogen bonds between A6 and the primer terminus T17. Thus, dATP stacks between A5 and A6 during the simulation. A similar nucleotide-stacking was also observed in pol λ's A(syn):dATP system. However, in pol λ, a positively-charged residue (Lys273) near A5 attracts A5 further away from dATP and stabilizes the DNA backbone, thereby shifting the DNA backbone [47]. In contrast, pol μ's negatively-charged Glu173 at the corresponding position “pushes” A5 back and keeps the DNA backbone near to its original position. As a result, the following shift of DNA backbone in pol λ's A(syn):dATP system does not occur in pol μ's A:dATP or A(syn):dATP systems.

**Figure 3:**
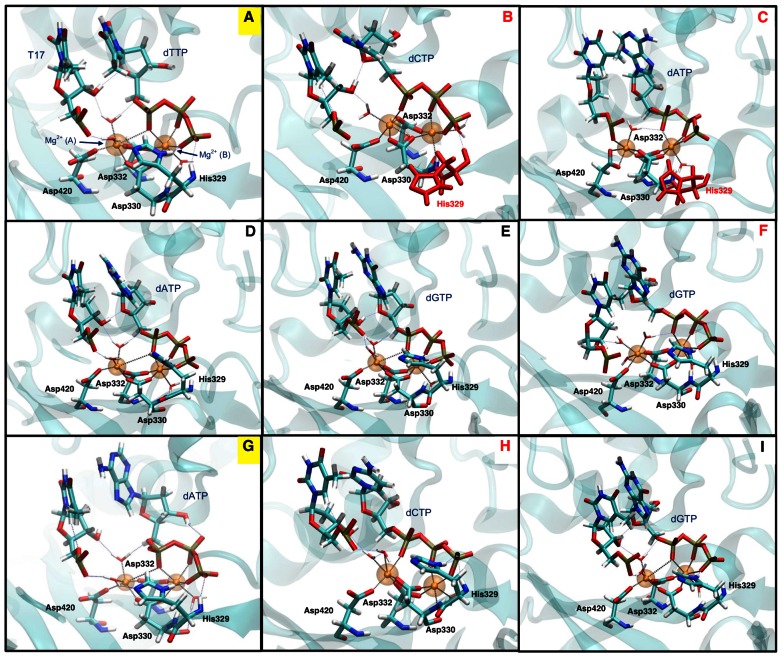
Representative active site arrangements from all pol μ cognate and non-cognate systems. (A) A:dTTP cognate system; (B) A:dCTP non-cognate system; (C) A:dATP non-cognate system; (D) A(syn):dATP non-cognate system; (E) A:dGTP non-cognate system; (F) A(syn):dGTP non-cognate system; (G) T:dATP cognate system; (H) T:dCTP non-cognate system; (I) T:dGTP non-cognate system. The two cognate systems (A and G) are labeled in yellow. Systems with significantly distorted active site compared to cognate A:dTTP system are titled in red. Flipped His329 in A:dATP and A:dCTP systems are marked as red. Bold dashed lines indicate coordination around Mg^2+^; thin dashed lines indicate hydrogen bonds. Mg^2+^ (A), catalytic ion; Mg^2+^ (B), nucleotide-binding ion.

The geometry of the active-site conformation in each system is shown in Fig. 3, and the critical distances in the active site are summarized in Table 1. The cognate A:dTTP and T:dATP systems share a similar active-site conformation: two water molecules coordinate with the catalytic Mg^2+^ ion (A). Mg^2+^ (A) connects with the primer terminus through two water molecules, and connects with the incoming nucleotide both directly and through a water molecule. Thus, the active site is relatively tight and appears ready for the chemical reaction. In the A:dGTP, A(syn):dATP, and T:dGTP non-cognate systems, few rearrangements in the active-site geometry occur. Mg^2+^ (A) connects to both the incoming nucleotide and the primer terminus through two water molecules, and the catalytic aspartate residues remain in their active conformation. The T:dCTP system has a similar active-site geometry in the beginning of simulation, but after 75 ns, O1A in the dCTP shifts away from the nucleotide-binding Mg^2+^ ion (B). After the shift, Mg^2+^ (B) coordinates with O in Asp330. Other coordination interactions in the T:dCTP system remains the same as the cognate system.

**Table 1:** Critical active-site distance in cognate and non-cognate systems.

Distance (Å)^a^	A:dTTP	A:dCTP	A:dATP	A(syn): dATP	A:dGTP	A(syn): dGTP	T:dATP	T:dCTP	T:dGTP
dNTP(Pα) - T17(O3′)	5.28±0.27	4.70±0.34	**8.06±0.32**	5.46±0.31	5.50±0.29	**8.44±0.30**	5.31±0.30	5.67±0.34	5.36±0.26
Mg^2+^ (A) - Mg^2+^ (B)	4.13±0.10	***3.62±0.15***	**4.43±0.09**	4.08±0.11	4.07±0.10	**4.43±0.07**	4.08±0.11	4.11±0.12	4.08±0.11
Mg^2+^ (A) - Asp330(OD2)	1.80±0.04	1.89±0.06	1.84±0.05	1.80±0.03	1.80±0.04	1.83±0.05	1.80±0.04	1.79±0.04	1.81±0.04
Mg^2+^ (A) - Asp332(OD1)	1.82±0.04	1.84±0.04	1.86±0.05	1.83±0.04	1.83±0.04	1.84±0.04	1.82±0.04	1.83±0.05	1.82±0.04
Mg^2+^ (A) - Asp420(OD2)	3.84±0.10	3.59±0.17	***2.13±0.39***	3.77±0.14	3.83±0.12	3.70±0.15	3.86±0.10	3.85±0.09	3.86±0.09
Mg^2+^ (A) - Asp420(OD1)	1.79±0.04	1.87±0.05	1.87±0.05	1.80±0.04	1.79±0.04	1.84±0.04	1.79±0.04	1.79±0.04	1.79±0.03
Mg^2+^ (A) - dNTP(O1A)	3.36±0.16	***1.85±0.05***	**4.06±0.15**	3.35±0.18	3.30±0.20	**4.01±0.10**	3.32±0.18	3.25±0.44	3.32±0.15
Mg^2+^ (A) - T17(O3′)	4.48±0.15	4.64±0.19	**6.75±0.25**	4.30±0.19	4.46±0.16	4.52±0.24	4.48±0.18	4.33±0.15	4.43±0.17
Mg^2+^ (B) - Asp330(OD1)	1.86±0.05	1.89±0.06	1.89±0.05	1.84±0.04	1.85±0.04	1.86±0.04	1.84±0.05	1.85±0.05	1.85±0.05
Mg^2+^ (B) - Asp330(O)	4.04±0.16	***2.13±0.13***	4.00±0.16	4.00±0.16	3.96±0.15	3.96±0.13	3.96±0.18	***2.35±0.21***	3.97±0.15
Mg^2+^ (B) - Asp332(OD2)	1.87±0.05	1.83±0.04	1.86±0.05	1.86±0.05	1.85±0.05	1.87±0.04	1.86±0.05	1.85±0.04	1.86±0.05
Mg^2+^ (B) - dNTP(O1B)	1.96±0.07	1.85±0.05	1.95±0.08	1.93±0.07	1.94±0.08	1.95±0.08	1.94±0.07	1.94±0.08	1.93±0.07
Mg^2+^ (B) - dNTP(O1A)	1.93±0.07	**3.48±0.12**	1.94±0.08	1.92±0.06	1.91±0.06	1.92±0.06	1.91±0.06	**3.68**±**0.14**	1.92±0.07
Mg^2+^ (B) - dNTP(O3G)	1.84±0.05	1.82±0.04	1.85±0.05	1.86±0.05	1.84±0.05	1.85±0.05	1.84±0.05	1.85±0.06	1.85±0.05

a Values are presented as mean ± standard deviation. Values in bold/bold italic are significantly different from (bold, larger than; bold italic, smaller than) the corresponding values in the cognate A:dTTP system. See Fig. S10 for more details on Mg^2+^ (A) - Mg^2+^ (B) and Mg^2+^ (A) - dNTP(O1A) distance data.

In a prior study of cognate pol μ systems, we found that His329 is the most sensitive residue to the absence or presence of incoming nucleotide. Its conformational change triggers the flip of the catalytic aspartate residue Asp330, thus contributing to the assembly of the active site [29]. In the A:dCTP system, His329 flips to an alternative conformation within 10 ns [Fig. S3(a)]. In the new conformation, His329 does not fully “open” to the inactive conformation, though still interrupts binding with dCTP. His329 further flips to its inactive “open” conformation but then flips back to the alternative conformation. Following the flip of His329, Asp330 rotates to an alternative conformation, where both OD1 and OD2 on Asp330 coordinate with Mg^2+^ (A). As a result, Mg^2+^ (A) coordinates with only one water molecule instead of two, and its connection with the primer terminus through water molecules weakens. Interestingly, the distance between and O1A on dCTP is significantly smaller than that in cognate A:dTTP system. However, O1A in the dCTP shifts away from Mg^2+^ (B), and Mg^2+^ (B) coordinates with O in Asp330, just as in the T:dCTP system.

In the A:dATP system, His329 also flips, but this is only followed by a slight rotation of Asp330 [at an 80% significance level, Fig. S3(b)]. The coordination around Mg^2+^ (B) remains the same. Asp420 rotates toward Mg^2+^ (A), and Mg^2+^ (A) coordinates with both OD1 and OD2 on Asp420. Due to attraction by Asp420, Mg^2+^ (A) shifts away from dATP, no longer able to directly coordinate with O1A on dATP, though it still interacts with O1A through a water molecule. Though Mg^2+^ (A) directly coordinates with the primer terminus T17, the distance between Mg^2+^ (A) and O3′ in T17 is significantly larger than that in the cognate system. In fact, the distance between O3′ in T17 and Pα in dATP is more than 8 Å (compared to ∼5 Å in the cognate system, Fig. S4), significantly larger than the optimal distance for chemical reaction. Again, distortion in the active site in the A:dATP system can be correlated to inactivity.

The A(syn):dGTP system also has a significantly larger O3′ - Pα distance. Like in A:dATP system, Mg^2+^ (A) also deviates from O1A in the incoming nucleotide, interacting with it only through a water molecule. Three water molecules coordinate with Mg^2+^ (A) instead of two in the cognate system. Because the third water molecule coordinate with neither the primer terminus nor the incoming nucleotide, interactions within the active site weaken overall. The three aspartate residues and His329 all remain in their active conformation.

In the A:dCTP, A:dATP, and A(syn):dGTP systems, water-mediated hydrogen bonds are generally weaker than direct hydrogen bonds in cognate systems. Therefore, active sites in those non-cognate systems have weaker internal interactions and thus may be more likely to deform.

We observe that even in the cognate system, the crucial O3′ - Pα distance (∼5 Å) appears to be longer than that is required for the chemical reaction (∼3 Å) [70], and also longer than the O3′ - Pα distance in the crystal structure (∼4 Å). Similar observations have been noted and discussed for various pol X family members [33], [36]. Such deviations likely occur because of the imperfection of force fields. For example, the energetics of divalent ions like Mg^2+^ are considered in the van der Waals (described by the phenomenological Lennard-Jones potential) and Coulombic interactions. Thus, while data generated for divalent ions with these force fields are generally useful and informative, ligand/ion distances may differ from those observed in high resolution x-ray crystal structures. Nonetheless, because our study focuses on general trends in Mg^2+^ ion coordination and involves systematic comparisons of the trends among closely-related systems, the above limitations are acceptable. Recent crystallographic studies also reveal that the O3′ - Pα distance may be much longer than the expected value [51].

In all the cognate and non-cognate systems we studied, the sugar puckers at the upstream primer and the dNTP remain in the C2′-endo state during our simulations.

### Nucleotide-binding Pocket Residues as “Gate-keepers”

**Figure 4:**
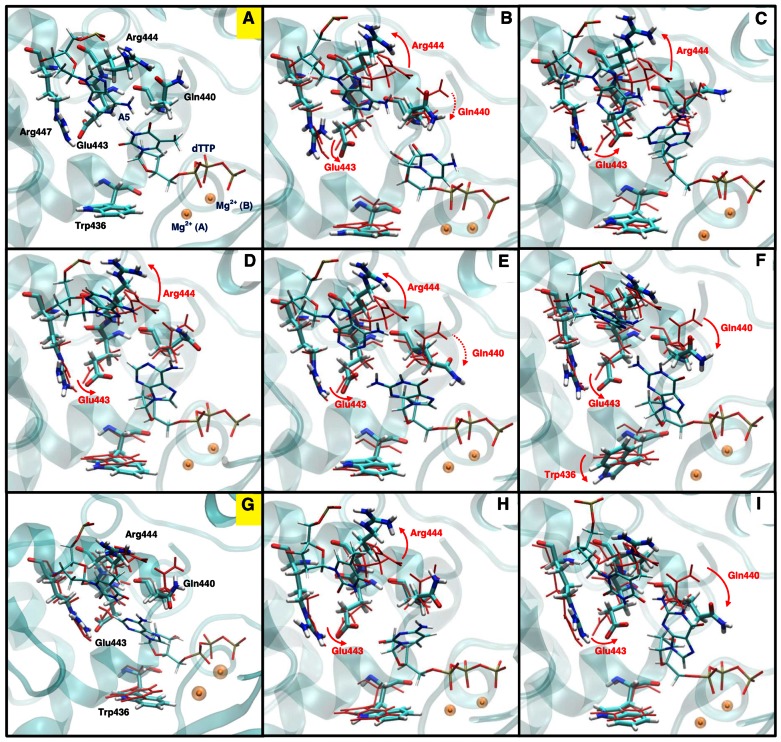
Representative conformations of nucleotide-binding pocket from all cognate and non-cognate systems. (A) A:dTTP cognate system; (B) A:dCTP non-cognate system; (C) A:dATP non-cognate system; (D) A(syn):dATP non-cognate system; (E) A:dGTP non-cognate system; (F) A(syn):dGTP non-cognate system; (G) T:dATP cognate system; (H) T:dCTP non-cognate system; (I) T:dGTP non-cognate system. The two cognate systems (A and G) are labeled in yellow. Only residues with conformational changes are labeled in non-cognate systems for clarity. Dashed arrows for Gln440 in A:dCTP and A:dGTP system indicate a tendency of shifting, yet not significantly. Mg^2+^ (A), catalytic ion; Mg^2+^ (B), nucleotide-binding ion.

Further rearrangements occur in the non-cognate systems that increase active-site disorder. A summary of residue rearrangements involved in each non-cognate system are provided in Table 2 and Fig. 4. In the cognate system of pol μ, a cognate incoming nucleotide triggers the rotation of Gln440 and Glu443, and this “loosens” the nucleotide-binding pocket and helps accommodate the incoming nucleotide. When an non-cognate nucleotide is present, Glu443 flips to an inactive state, thus “tightening” the nucleotide-binding pocket and deactivating the active site. Following the flip, Glu443 may interact with the non-cognate nucleotide through water molecules in several non-cognate systems, though the water-bridged interactions are dynamic. Interestingly, after the flip, the distance between Glu443 and the nucleotide does not decrease (Fig. S5). Thus, the deactivation effect of Glu443's flip may be due to an electrostatic effect rather than the steric hindrance. We discuss this further in the next section. We hypothesize that a mutation of Glu443 to a residue with similar length but neutral charge (for example, methionine) may reduce the fidelity of pol μ. Such an E443M substitution may reduce the catalytic ability for both cognate and non-cognate systems, but the cognate system may be affected more. Thus, the fidelity of the E443M mutant may decrease. This hypothesis may be tested by further experimental and computational studies.

**Table 2:** Summary of key residues' motion in active-site and nucleotide-binding pocket.

Key residue^a^	A:dCTP	A:dATP	A(syn):dATP	A:dGTP	A(syn):dGTP	T:dCTP	T:dGTP
His329	**Twisted**	**Twisted**	Active	Active	Active	Active	Active
Asp330	**Twisted**	**Slightly twisted**	Active	Active	Active	Active	Active
Asp420	Active	**Twisted**	Active	Active	Active	Active	Active
Trp436	Mainly active	Active	Active	Active	**Twisted**	Active	Active
Gln440	**Shifting**	Active	Active	**Shifting**	**Shifted**	Active	**Shifted**
Glu443	**Flipped**	**Flipped**	**Flipped**	**Flipped**	**Flipped**	**Flipped**	**Flipped**
Arg444	**Flipped**	**Flipped**	**Flipped**	**Flipped**	Active	**Flipped**	Active

a Key residue in bold shows different behavior compared to that in the cognate A:dTTP/T:dATP system.

The motion of Gln440 is more flexible. Without dNTP, Gln440 flips to its inactive form and binds to the primer terminus. In the non-cognate systems, Gln440 cannot bind to the primer terminus because of the hindrance, and therefore it cannot reach a stable inactive nor active state. When we plot the distance between the center of mass of Gln440 and the center of mass of dNTP in Fig. S6(a), we see that in the A(syn):dGTP and T:dGTP systems, Gln440 is significantly closer to the incoming nucleotide than in the cognate systems (computed with the data from the last 40 ns, at a confidence level of 90% and 85%, respectively). In the A:dCTP and A:dGTP systems, Gln440 also displays a tendency to shift towards dNTP. The average distance between Gln440 and dNTP decreases for 0.44 Å and 0.62 Å over the simulation in A:dCTP and A:dGTP systems, respectively. In contrast, overall change is only 0.02 Å in the cognate A:dTTP system. Therefore, Gln440 also participates in “tightening” the active site by shifting towards the non-cognate nucleotide. These two residues are not conserved in pol β or pol λ, and thus must be unique to pol μ function.

As its corresponding residue Arg514 in pol λ, Arg444 in pol μ mainly helps stabilize the template base at the gap through stacking interactions [29], [36]. It also stabilizes Gln440 in its active conformation by hydrogen bonding to it. However, unlike Arg514 in pol λ, Arg444 in pol μ does not participate in the conformational change of active site upon binding a cognate nucleotide. Because of the distortion in the mispaired bases, the stacking interactions are interrupted in non-cognate systems. Therefore, Arg444 flips away from the active site in all non-cognate systems except A(syn):dGTP and T:dGTP [Fig. S7(a)]. The flip of Arg444 increases the flexibility of Gln440 because the hydrogen bond between Arg444 and Gln440 breaks. However, the flip of Arg444 does not necessarily cause the shift of Gln440 towards the non-cognate dNTP. In addition, because Arg444 binds to the backbone of the template base A5, its conformational change may also induce the shift of A5 away from the active site.

Trp436 in pol μ is analogous to Phe272 in pol β, or Phe506 in pol λ, residues that initiate DNA or subdomain motions through a flip during the conformational transition of the polymerase complex [33],[36]. When pol μ incorporates a cognate nucleotide, no significant motion of Trp436 is observed because DNA or subdomain motion is not part of pol μ's conformational pathway. However, in the A(syn):dGTP system, Trp436 rotates its indole ring towards dGTP [Fig. S7(b)]. The rotation of Trp436 limits the space in the active site and pushes the dGTP away from the active site, thereby also “tightening” the nucleotide-binding pocket. We observe a similar rotation occasionally in the A:dCTP system toward the end of the simulation.

Arg447 in pol μ is analogous to Arg283 in pol β and Arg517 in pol λ, both important for checking the cognate base-pairing [47], [71]–[73]. Arg517 in pol λ is also crucial in pol λ's ability to accommodate frame-shifted DNA [74]. This arginine binds to both the base at the gap and the one pairing with primer terminus in the template DNA, and stabilizes DNA in the closed form of complex. However, in pol β and pol λ, this binding is sensitive to the incoming nucleotide context. When an non-cognate nucleotide is present and the active site is distorted by abnormal base pairing, fewer direct hydrogen bond interactions between Arg283 (in pol β) and Arg517 (in pol λ) with the DNA occur [41], [47]. This leads to the poor stabilization of the DNA template bases, which incurs further rearrangements of incoming nucleotides and/or shift of DNA backbone. In contrast, in pol μ, the binding of Arg447 to DNA is not affected significantly by the non-cognate nucleotides. The direct hydrogen bonding of Arg447 - A6:N3 and Arg447 - A6:O4′, as well as Arg447 - A6:O1P interaction through a water molecule, are present in all non-cognate systems. Arg447 - A5:N3 or Arg447 - T5:O2 interaction is also present in all systems except A(syn):dATP and A(syn):dGTP, where the *syn* conformation of A5 keeps N3 away from Arg447. In T:dGTP system, Arg447 - T5:O2 interaction is not stable. The hydrogen bond between them does not form until after 90 ns, and deforms near the end of our 120 ns simulation [Fig. S6(b)]. However, in the A(syn):dGTP and T:dGTP systems, Arg444 does not flip and stacks with A5 or T5. Thus, the mispaired bases are still stable, and further rearrangements or motions caused by the lack of Arg447 interactions are not observed. This may suggest that pol μ's active site is more flexible than those in pol β and pol λ, so it might better accommodate the non-cognate nucleotide without breaking Arg447/DNA interactions. The flexibility of active site also supports the observation that pol μ can accommodate and insert ribonucleotides in the active site [75].

Motions of “Gate-keeper” residues, namely the flip of Glu443, shifting of Gln440, flip of Arg444, and rotation of Trp436, are not observed in our modeled cognate T:dATP system. This further confirms that “gate-keeper” residues can help discriminate non-cognate nucleotides from cognate ones and thus may have a significant role in controlling the fidelity of pol μ.

**Figure 5:**
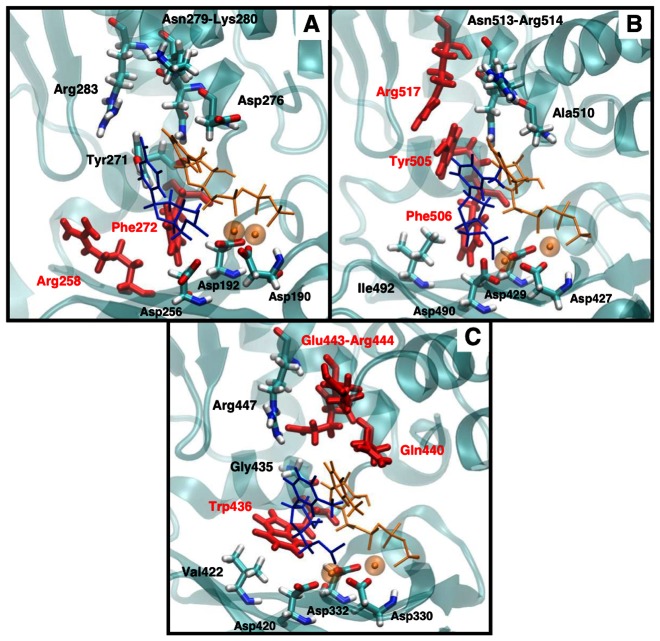
Comparison of “gate-keeper” residue locations in pol X family members. (A) pol β; (B) pol λ; (C) pol μ. “Gate-keeper” residues in each polymerase are colored red; incoming nucleotide and Mg^2+^ in orange; and upstream primer in blue. See also Table S1 for distance data.

Three of the four “gate-keeper” residues in pol μ (Gln440, Glu443, and Arg444) are located apart from the upstream primer (>8 Å) and near the downstream primer; Trp436, which is near the upstream primer, functions as “gate-keeper” residue in only one non-cognate system (A:dGTP). In comparison to other X-family members, both “gate-keeper” residues in pol β (Arg258 and Phe272), as well as two of three “gate-keeper” residues in pol λ (Tyr505 and Phe506), are located near the upstream primer (<6.5 Å, Fig. 5). This difference may be related to the fact that pol μ can incorporate and insert the incoming nucleotide when the upstream primer is not paired. That is, pol μ may be less sensitive to changes around the upstream primer. Residue flexibility differences when the upstream primer is not paired may be interesting to explore in future computational and experimental studies of pol μ.

Using the number of dNTP and protein residue changes in Table 1 and Table 2, we suggest the following sequence for difficulty of nucleotide incorporation by pol μ: T:dGTP<A(syn):dATP<T:dCTP<A:dGTP<A(syn):dGTP<A:dCTP<A:dATP (T:dGTP is the easiest to incorporate and A:dATP is the most difficult). This sequence agrees with the observed trends in the reaction kinetics data for nucleotide insertion [17], [18], as summarized in Table 3, with the exception of A:dGTP, which may depend sensitively on the surrounding sequence [76]. For example, kinetic data obtained with another DNA sequence [19] suggests a different trend: T:dCTP<A:dCTP<T:dGTP<A:dATP<A:dGTP. Another possible explanation for our observing greater difficulty in the A:dGTP mispair relative to T:dCTP and T:dGTP while kinetic data indicate that A:dGTP is more favorable is that T:dCTP and T:dGTP mispairs are less favorable overall for chemical reaction following the conformational changes, as discussed below.

**Table 3:** Base substitution fidelity of pol μ taken from ref 17.

Enzyme complex	Maximum reaction velocity, V_max_ (s^−1^)	Incorporation efficiency, V_max_/K_m_ (M^−1^⋅s^−1^)	Fidelity^a^
A:dTTP	0.058	3.1×10^4^	1
A:dATP	0.00060	1.7	5.4×10^−5^
A:dCTP	Not detected	6.3^b^	2.1×10^−4^
A:dGTP	0.036	1.1×10^3^	3.6×10^−2^
T:dATP	0.054	7.0×10^3^	1
T:dCTP	Not detected	5.0^b^	7.1×10^−4^
T:dGTP	0.00055	7.4	1.0×10^−3^

a Fidelity = (V_max_/K_m_)_non-cognate_/[(V_max_/K_m_)_cognate_+(V_max_/K_m_)_non-cognate_].

b The V_max_/K_m_ value was determined by the slope of the initial velocity.

### Altered Electrostatic Potential around dNTP

**Figure 6:**
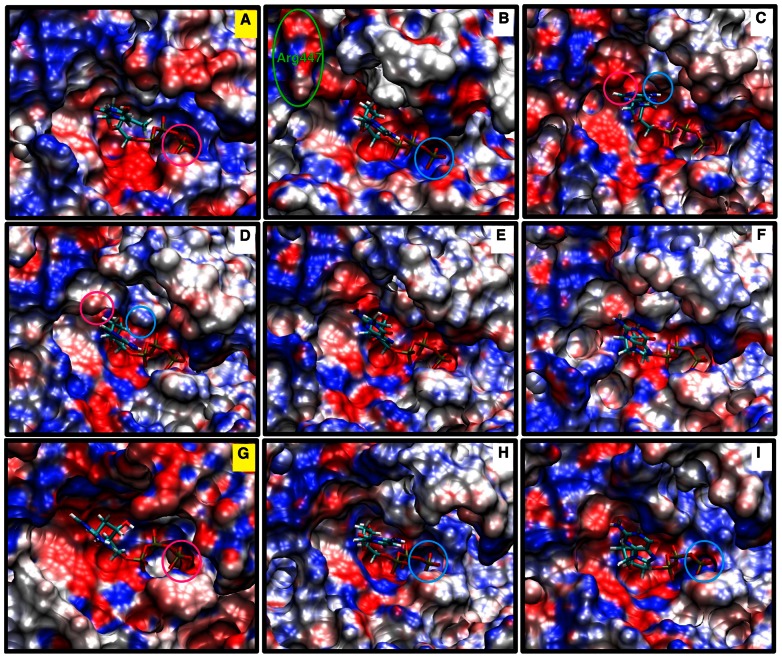
Electrostatic potential surfaces of the pol μ active site with cognate or non-cognate incoming nucleotides. (A) A:dTTP cognate system; (B) A:dCTP non-cognate system; (C) A:dATP non-cognate system; (D) A(syn):dATP non-cognate system; (E) A:dGTP non-cognate system; (F) A(syn):dGTP non-cognate system; (G) T:dATP cognate system; (H) T:dCTP non-cognate system; (I) T:dGTP non-cognate system. The two cognate systems (A and G) are labeled in yellow.

We further examine in Fig. 6 the electrostatic potential of pol μ's active site with the cognate A:dTTP and T:dATP base pairs and various non-cognate systems. Unfavorable protein/dNTP interactions emerge in the non-cognate systems that destabilize the dNTP. Though subtle differences exist, the active site in pol μ's cognate A:dTTP and T:dATP systems have mainly negative (red) electrostatic potential, whereas the non-cognate systems have more neutral (white) or positive (blue) electrostatic potentials. Interestingly, for pol λ, changes in electrostatic potential are also observed, but in an opposite way: more neutral or positive for the cognate system, and more negative for the non-cognate system [47]. The changes in electrostatics environments suggest altered interactions within the active site, which in turn affect active-site rearrangements.

In the A:dCTP system, Arg447 (green circle in Fig. 6B) appears in a negatively charged region, thus its stabilization effect to DNA template base A5 and A6 weakens, which in turn may destabilize the dCTP and primer terminus pairing with A5 and A6. In A:dATP and A(syn):dATP system, following the flip of Glu443, the region near N1 and N3 atoms of dATP becomes more negative (pink circle in Fig. 6C and 6D), while the region near the amino group of dATP is relatively more positive (cyan circle in Fig. 6C and 6D). These two disruptive forces together destabilize dATP and drive it towards the primer terminus direction, allowing dATP to stack between A5 and A6. Moreover, in the A:dCTP, T:dCTP, and T:dGTP systems, the end of the phosphate group on dNTP falls into a mainly positive region (cyan circle in Fig. 6B, 6H, and 6I, compared to pink circle in the cognate systems, Fig. 6A and 6G), which is unfavorable for the proton transfer reaction to follow. Therefore, not only does the altered electrostatic potential around dNTP disturb the conformational rearrangements in active site, but it also may it affect the chemical step after the conformational changes.

### Reduced Correlated Motions within Pol μ Complex

In prior work we studied the coupled conformational changes within polymerase complex upon binding a cognate or non-cognate incoming nucleotide for pol λ, pol β, and pol X [52]. Similar coupled motions within the same subdomain, among different subdomains, and between protein and DNA/dNTP, were revealed across the X family. Even within the same subdomain, the coupled regions can be distant in space. These correlated motions together drive the polymerase towards its active form. When an non-cognate nucleotide is bound, such correlated motions decrease.

**Figure 7:**
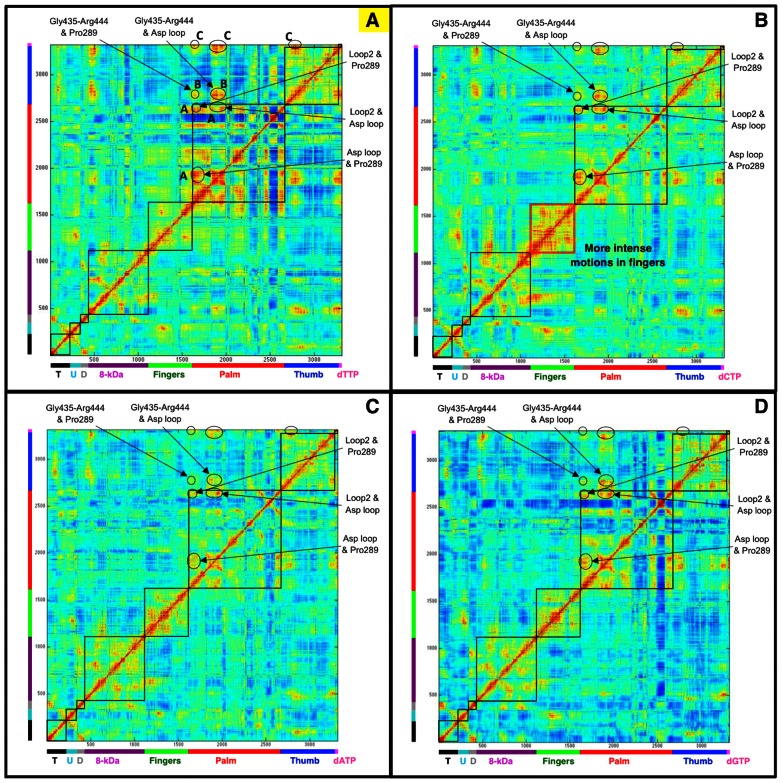
Covariance matrix for protein/DNA/dNTP heavy atoms in A:dTTP, A:dCTP, A:dATP, and A:dGTP pol μ systems. (A) A:dTTP cognate system; (B) A:dCTP non-cognate system; (C) A:dATP non-cognate system; (D) A:dGTP non-cognate system. T, DNA template strand; U, DNA upstream primer strand; D, DNA downstream primer strand. Colors: black (template), cyan (upstream primer), silver (downstream primer), purple (8-kDa domain), green (fingers), red (palm), blue (thumb), and magenta (dNTP). The cognate system (A) is labeled in yellow. Note the more intense interactions within fingers in A:dCTP system. See also Fig. S8 for the difference matrices.

Correlated motions in pol μ system are shown in Fig. 7. The cognate system of pol μ displays a similar network of coupled motions as pol β and pol λ, but with fewer interactions. Specifically, within the palm subdomain, correlated motions are observed among three regions as follows (region A in Fig. 7): a conserved X-family loop [77] (Thr314-Thr336) containing two of the three catalytic aspartates (Asp330 and Asp332) with a region (Thr288-Val290) near the finger including Pro289 (its analogous residue Arg149 in pol β or Arg346 in pol λ binds with the incoming nucleotide, though Pro289 in pol μ does not has such binding ability); the Asp loop with the Loop 2 (Ala407-Lys417) that is apart from the active site; and the Pro289 region with the Loop 2. Gly435-Arg444 in the thumb that includes the nucleotide-binding pocket residues Gly435, Trp436, Gln440, Glu443, and Arg444, also correlate with the Asp loop and Pro289 region in the palm (region B). All these regions in the palm and thumb are further correlated to the dTTP (region C).

Non-cognate systems of pol μ generally have less correlated motions than the cognate system. In all the three non-cognate systems, the correlated motions between Gly435-Arg444 and the dTTP, and those between Gly435-Arg444 and Pro289 region are greatly reduced or missing. The A:dGTP system has the least changes of coupled motions, and is most similar to the cognate A:dTTP system. In the A:dCTP system, more intense motions correlated within the fingers are observed. The correlated motions between the polymerase fingers and 8-kDa domain, and between the fingers and DNA also increase. These motions may suggest that pol μ requires more conformational rearrangements in the finger when accommodating dCTP. With these additional conformational changes, pol μ deviates from its active conformation. The A:dATP non-cognate system is the most different of the three, compared to the cognate system. Correlated motions between the Asp loop and Gly435-Arg444 are reduced significantly. Because both the Asp loop and Gly435-Arg444 are within the active site, the reduced interactions among active-site residues largely hamper the orchestration of cooperative events to reach at an optimal active-site conformation. Almost all other correlated motions in the A:dATP system also appear reduced. The limited correlated motions suggest that A:dATP system remains in an inactive.

Overall, our correlated motion analysis suggests an order of A:dTTP≈A:dGTP>A:dCTP>A:dATP for the degree of correlated motions. This order also agrees with the trend we suggested above from active-site distortion and key residue motion. We further present the difference matrices between the A:dTTP cognate system and the A:dATP/A:dCTP/A:dGTP non-cognate systems in Fig. S8. Within the three systems, the correlated motion of A:dGTP system is most similar to that of the cognate A:dTTP system, suggesting a more favorable active site for the following chemical step. This may explain the high misincorporation rate of A:dGTP observed in experiments (Table 3). These results provide further support for the hybrid conformational selection/induced-fit model for polymerases: before substrate binding, the polymerase/DNA complex adapts a series of possible conformations, and substrate binding stabilizes specific conformation. This inherent flexibility is evident from Fig. S9, which reveals the correlated motions when the substrate (dNTP) is absent. From this ensemble of conformations, in the A:dTTP cognate system, dTTP would selectively bind to a near-active conformation and guide the system into a fully active form as well as trigger required active-site changes. In the relatively active A:dGTP system, dGTP would also selectively bind to a near-active conformation with correlated motions similar to those in A:dTTP system. However, the suboptimal fit of dGTP within the active site induces active-site changes that differ from that in A:dTTP system. In the A:dCTP and A:dATP systems, the dNTPs bind to variable conformations of pol μ that deviate from the active forms; those tailored fits, however, hamper correlated motions that are essential for preparing the enzyme for subsequent catalysis.

## Conclusion

Our molecular dynamics simulations of pol μ cognate and non-cognate systems reveal significant differences in the active site and regarding the correlated motions upon binding an non-cognate nucleotide compared to a cognate substrate. The results suggest that, compared to pol β or pol λ, no significant changes in global motion of protein or DNA would occur for pol μ. His329 and Asp330 in the active site, as well as Trp436, Gln440, Glu443, and Arg444 in the nucleotide-binding pocket, play the role of “gate-keeper” in pol μ. These residues alter the electrostatic potential in the active site and trigger the distortion of active site when an non-cognate nucleotide is bound. Because most “gate-keeper” residues in pol μ are relatively far from the upstream primer, this fact may explain in part pol μ's ability to incorporate nucleotides when the upstream primer is not paired. Furthermore, in non-cognate systems, correlated motions within the complex are reduced. These results suggest that like other X-family polymerase, pol μ also fits in a hybrid conformational select/induced-fit model; the cognate substrate would bind to the active form and trigger active-site changes, while non-cognate substrates with relative high efficiency would bind to an active form but not trigger the following active-site changes, and non-cognate substrates with poor efficiency would bind to variable conformations. The degree of active-site geometry distortion determined from our simulations roughly parallels the kinetic data, suggesting a direct relation between active-site structural distortions and fidelity of pol μ. We also suggest experimentally testable predictions that mutation on pol μ's “gate-keeper” residues, like E443M, may reduce the fidelity of pol μ.

## Supporting Information

Figure S1 **Protein (A) and DNA (B) global motions in cognate and non-cognate systems.** Colors: red (A:dTTP), blue (A:dATP), cyan [A(syn):dATP], green (A:dCTP), pink (A:dGTP), purple [A(syn):dGTP], orange (T:dCTP), and black (T:dGTP). Only bases of DNA in A:dTTP system are shown for clarity. Note that the shift of A5/T5 does not occur large shift of DNA backbones. See also Fig. S2 for the shift of A5.
(TIF)

Figure S2 **Root mean standard deviation (RMSD) of A5 base in DNA template strand in selected systems.**
(TIF)

Figure S3 **Plots of dihedral angle data for His329 and Asp330.** (A) His329 (His329:CG - His329:CB - His329:CA - His329:C) in all cognate and non-cognate systems; (B) Asp330 (His329:C - Asp330:N - Asp330:CA - Asp330:CB) in A:dTTP, A:dATP, and A:dCTP systems.
(TIF)

Figure S4 **Critical distance of T17:O3′ - dNTP:Pα in all cognate and non-cognate systems.**
(TIF)

Figure S5 **Cluster analysis of Glu443 in all cognate and non-cognate systems.** Clustering is based on dihedral angle (Glu443:CA - Glu443:CB - Glu443:CG - Glu443:CD) and distance to the DNA (Glu443:CD - A5:C2 or T5:C2).
(TIF)

Figure S6 **Plots of distance data for Gln440 and Arg447.** (A) Distance between center of mass of Gln440 and dNTP in A:dTTP, A(syn):dGTP, and T:dGTP systems; (B) distance between Arg447:HH12 and A5:N3 or T5:O2 in all cognate and non-cognate systems.
(TIF)

Figure S7 **Plots of dihedral angle data for Arg444 and Trp436.** (A) Arg444 (Arg444:CG - Arg444:CB - Arg444:CA - Arg444:N) in all cognate and non-cognate systems; (B) Trp436 (Trp436:CD2 - Trp436:CG - Trp436:CB - Trp436:CA) in A:dTTP and A(syn):dGTP systems.
(TIF)

Figure S8 **Difference covariance matrix for protein/DNA heavy atoms in non-cognate systems compared to A:dTTP system.** (A) A:dATP; (B) A:dCTP; and (C) A:dGTP. Lighter color represents less difference compared to A:dTTP system. T, DNA template strand; U, DNA upstream primer strand; D, DNA downstream primer strand. Colors: black (template), cyan (upstream primer), silver (downstream primer), purple (8-kDa domain), green (fingers), red (palm), blue (thumb), and magenta (dNTP).
(TIF)

Figure S9 **Covariance matrix for protein/DNA heavy atoms in nucleotide-absent system of pol μ.** T, DNA template strand; U, DNA upstream primer strand; D, DNA downstream primer strand. Colors: black (template), cyan (upstream primer), silver (downstream primer), purple (8-kDa domain), green (fingers), red (palm), and blue (thumb).
(TIF)

Figure S10 **Plots of distance data of Mg^2+^.** (A) Distance between Mg^2+^ (A) and Mg^2+^ (B) in all cognate and non-cognate systems; (B) Distance between Mg^2+^ (A) and dNTP:O1A in all cognate and non-cognate systems.
(TIF)

Text S1 **Details on protonation states and correlated motion analysis.**
(PDF)

Table S1 **Distance between “gate-keeper” residues and the upstream primer in pol β, pol λ, and pol μ complexes.**
(PDF)

Table S2 **Protonation states of titratable side chains and phosphate groups in pol μ.**
(PDF)
