# Supplementary figures and images for: “Gate-keeper” Residues and Active-Site Rearrangements in DNA Polymerase μ Help Discriminate Non-cognate Nucleotides
Source: PLoS Comput Biol. 2013 May 23;9(5):e1003074. doi: 10.1371/journal.pcbi.1003074 (PMC3662701; doi:10.1371/journal.pcbi.1003074)

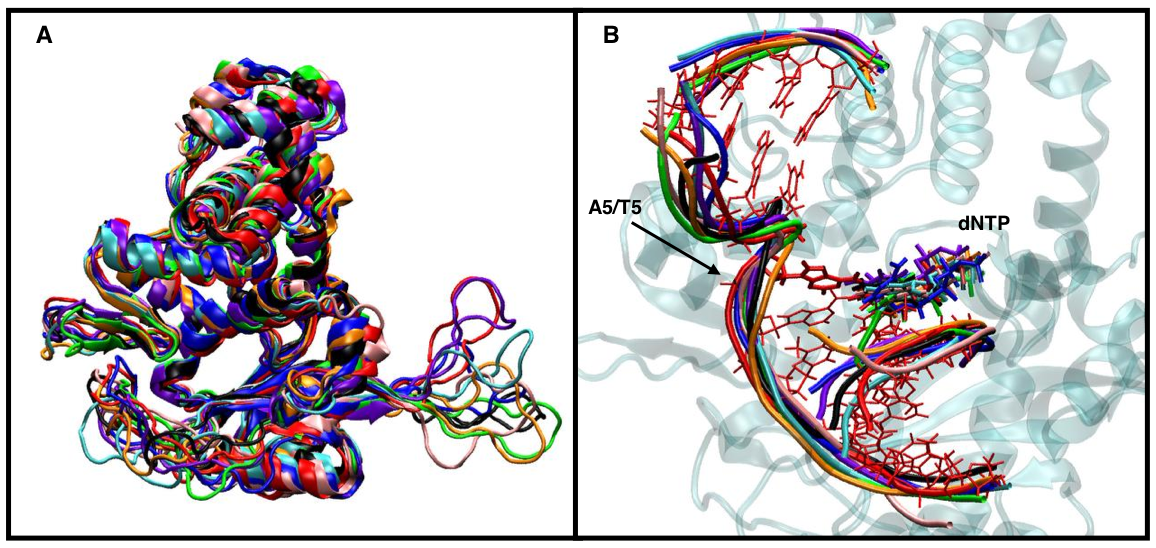

Supplement: Figure S1 — Protein (A) and DNA (B) global motions in cognate and non-cognate systems. Colors: red (A:dTTP), blue (A:dATP), cyan [A(syn):dATP], green (A:dCTP), pink (A:dGTP), purple [A(syn):dGTP], orange (T:dCTP), and black (T:dGTP). Only bases of DNA in A:dTTP system are shown for clarity. Note that the shift of A5/T5 does not occur large shift of DNA backbones. See also Fig. S2 for the shift of A5. (TIF) [file pcbi.1003074.s001.tif]

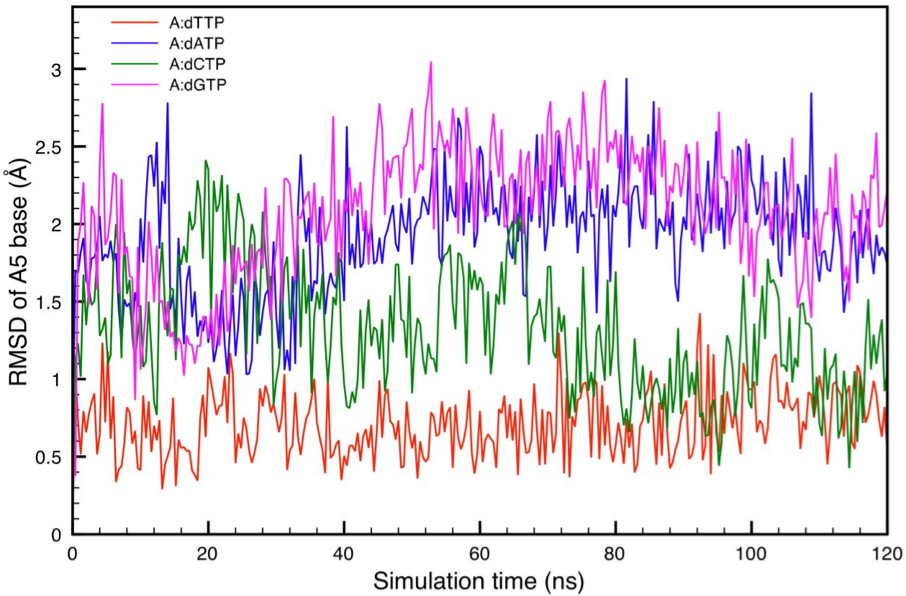

Supplement: Figure S2 — Root mean standard deviation (RMSD) of A5 base in DNA template strand in selected systems. (TIF) [file pcbi.1003074.s002.tif]

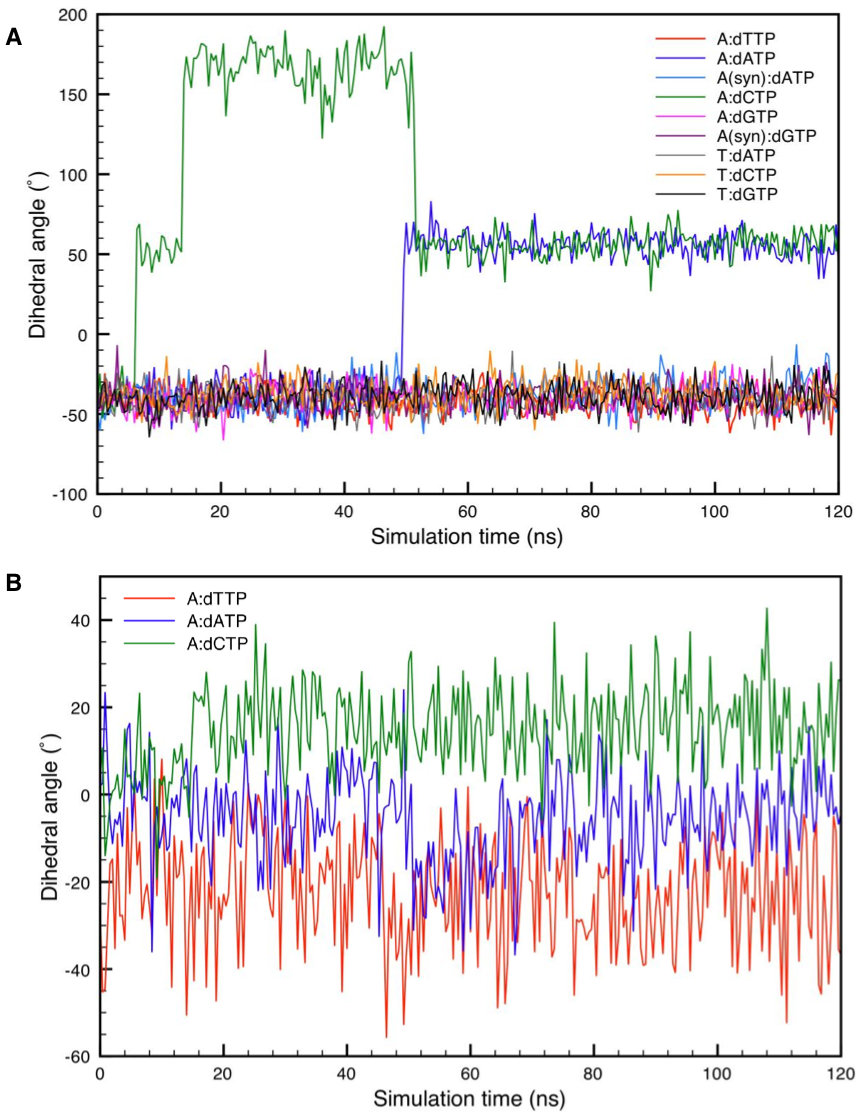

Supplement: Figure S3 — Plots of dihedral angle data for His329 and Asp330. (A) His329 (His329:CG - His329:CB - His329:CA - His329:C) in all cognate and non-cognate systems; (B) Asp330 (His329:C - Asp330:N - Asp330:CA - Asp330:CB) in A:dTTP, A:dATP, and A:dCTP systems. (TIF) [file pcbi.1003074.s003.tif]

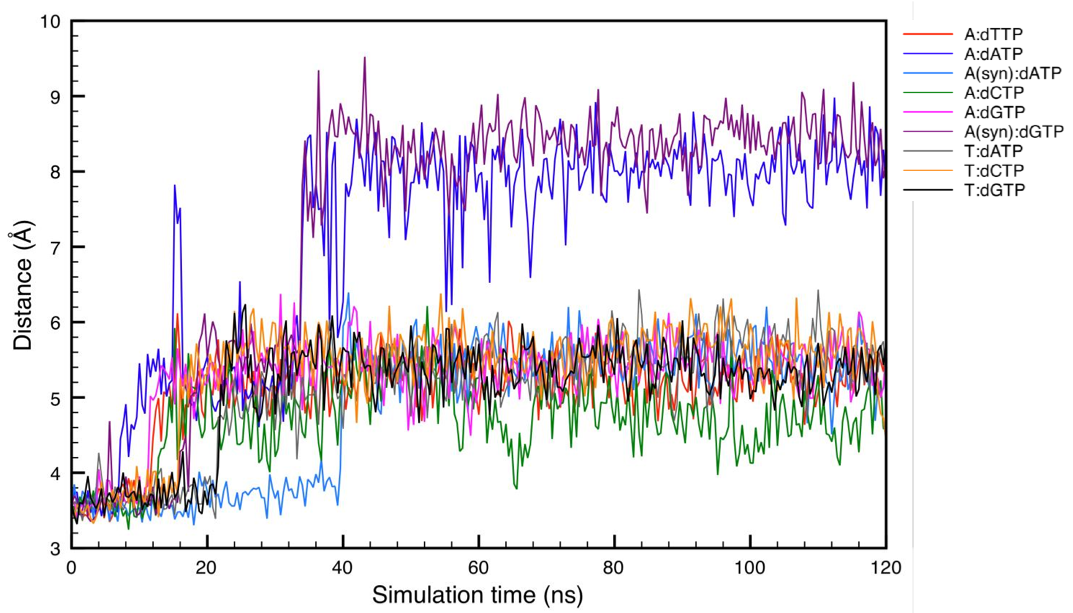

Supplement: Figure S4 — Critical distance of T17:O3′ - dNTP:Pα in all cognate and non-cognate systems. (TIF) [file pcbi.1003074.s004.tif]

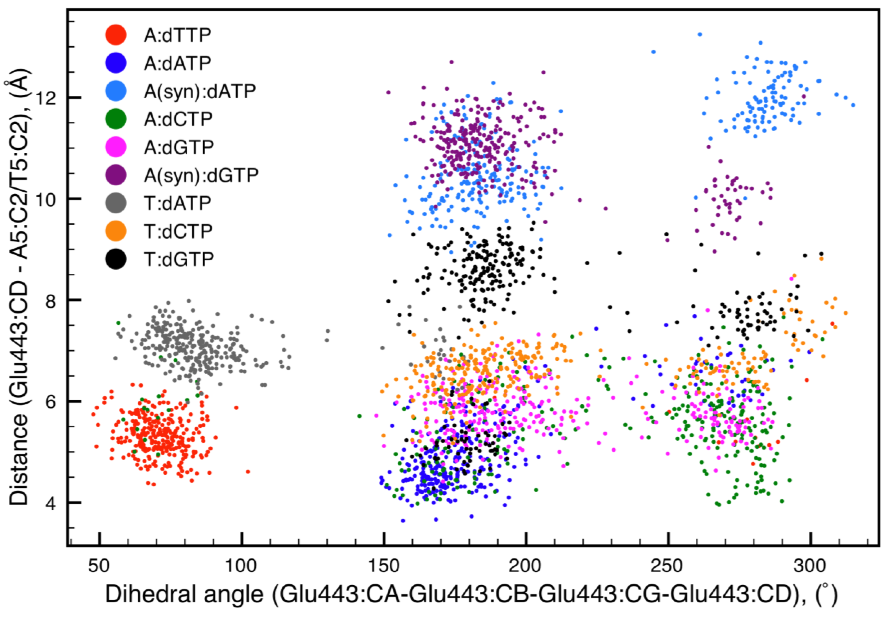

Supplement: Figure S5 — Cluster analysis of Glu443 in all cognate and non-cognate systems. Clustering is based on dihedral angle (Glu443:CA - Glu443:CB - Glu443:CG - Glu443:CD) and distance to the DNA (Glu443:CD - A5:C2 or T5:C2). (TIF) [file pcbi.1003074.s005.tif]

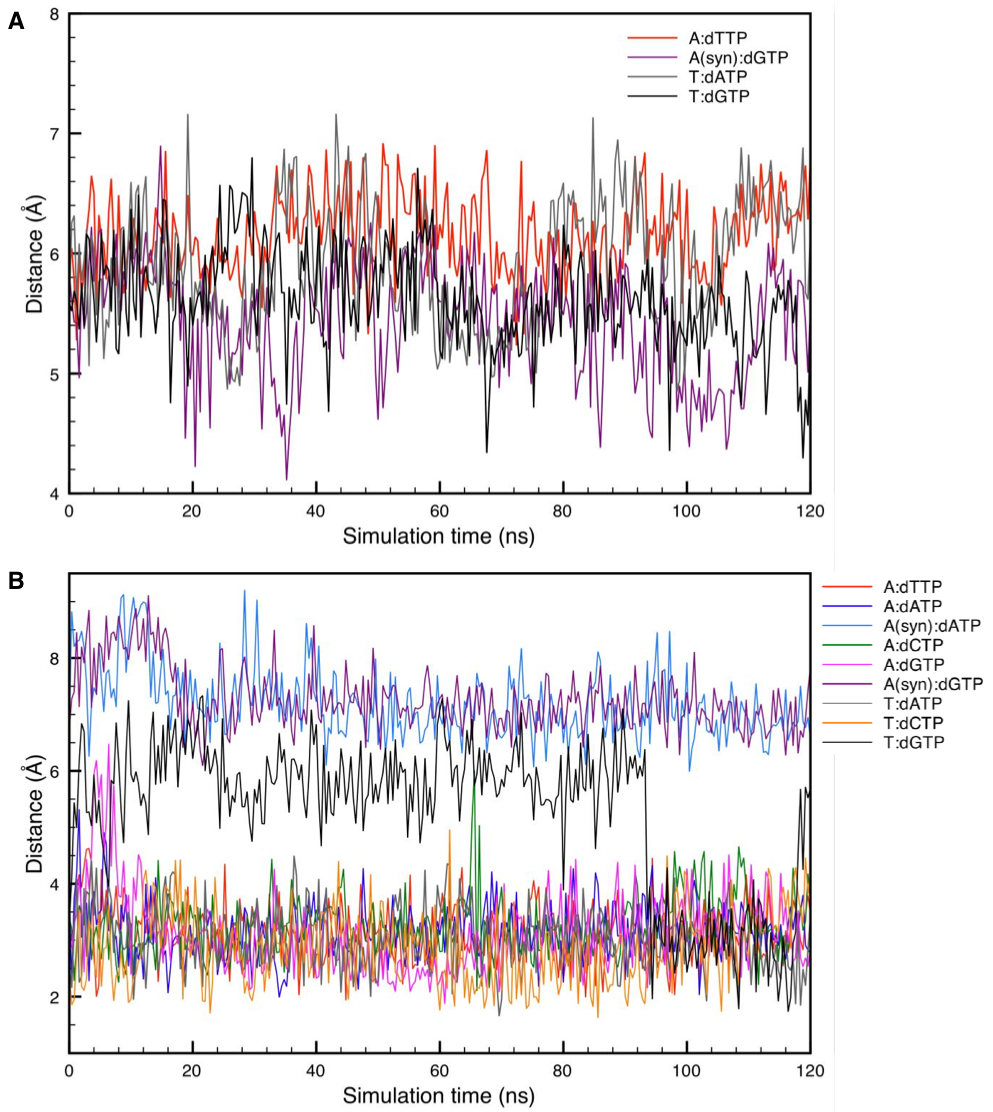

Supplement: Figure S6 — Plots of distance data for Gln440 and Arg447. (A) Distance between center of mass of Gln440 and dNTP in A:dTTP, A(syn):dGTP, and T:dGTP systems; (B) distance between Arg447:HH12 and A5:N3 or T5:O2 in all cognate and non-cognate systems. (TIF) [file pcbi.1003074.s006.tif]

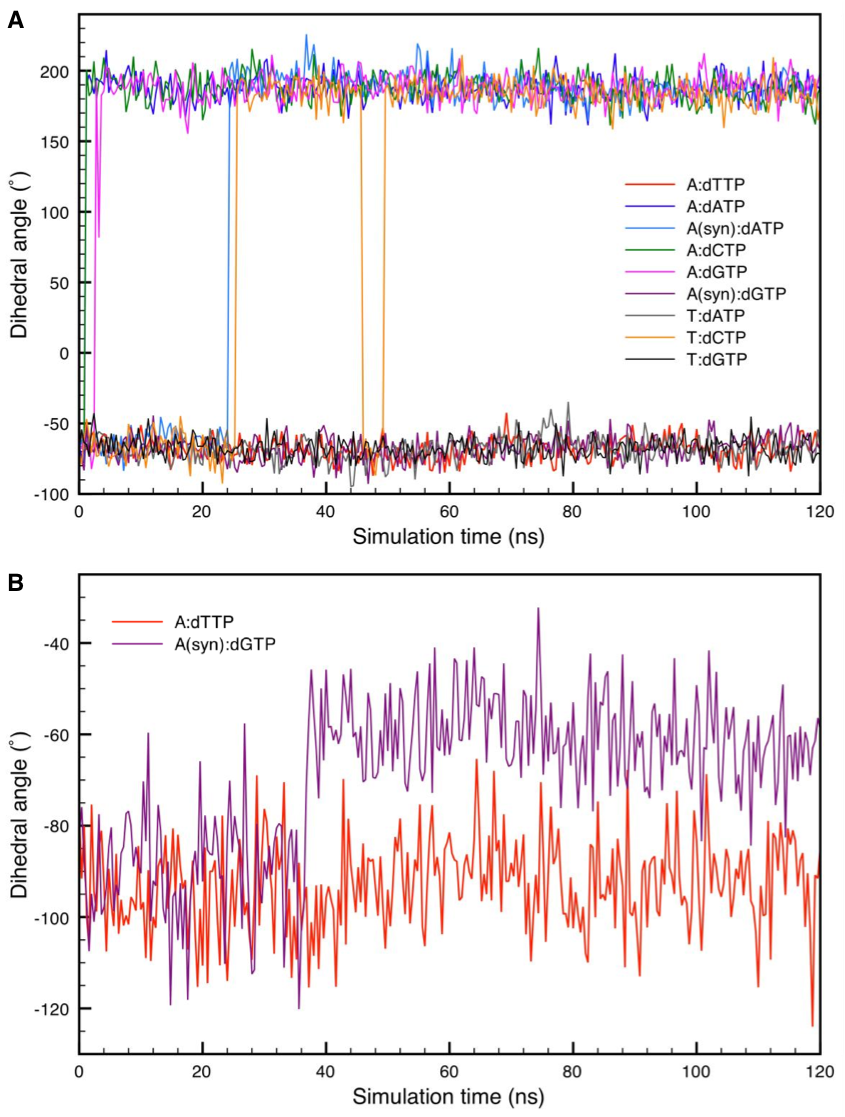

Supplement: Figure S7 — Plots of dihedral angle data for Arg444 and Trp436. (A) Arg444 (Arg444:CG - Arg444:CB - Arg444:CA - Arg444:N) in all cognate and non-cognate systems; (B) Trp436 (Trp436:CD2 - Trp436:CG - Trp436:CB - Trp436:CA) in A:dTTP and A(syn):dGTP systems. (TIF) [file pcbi.1003074.s007.tif]

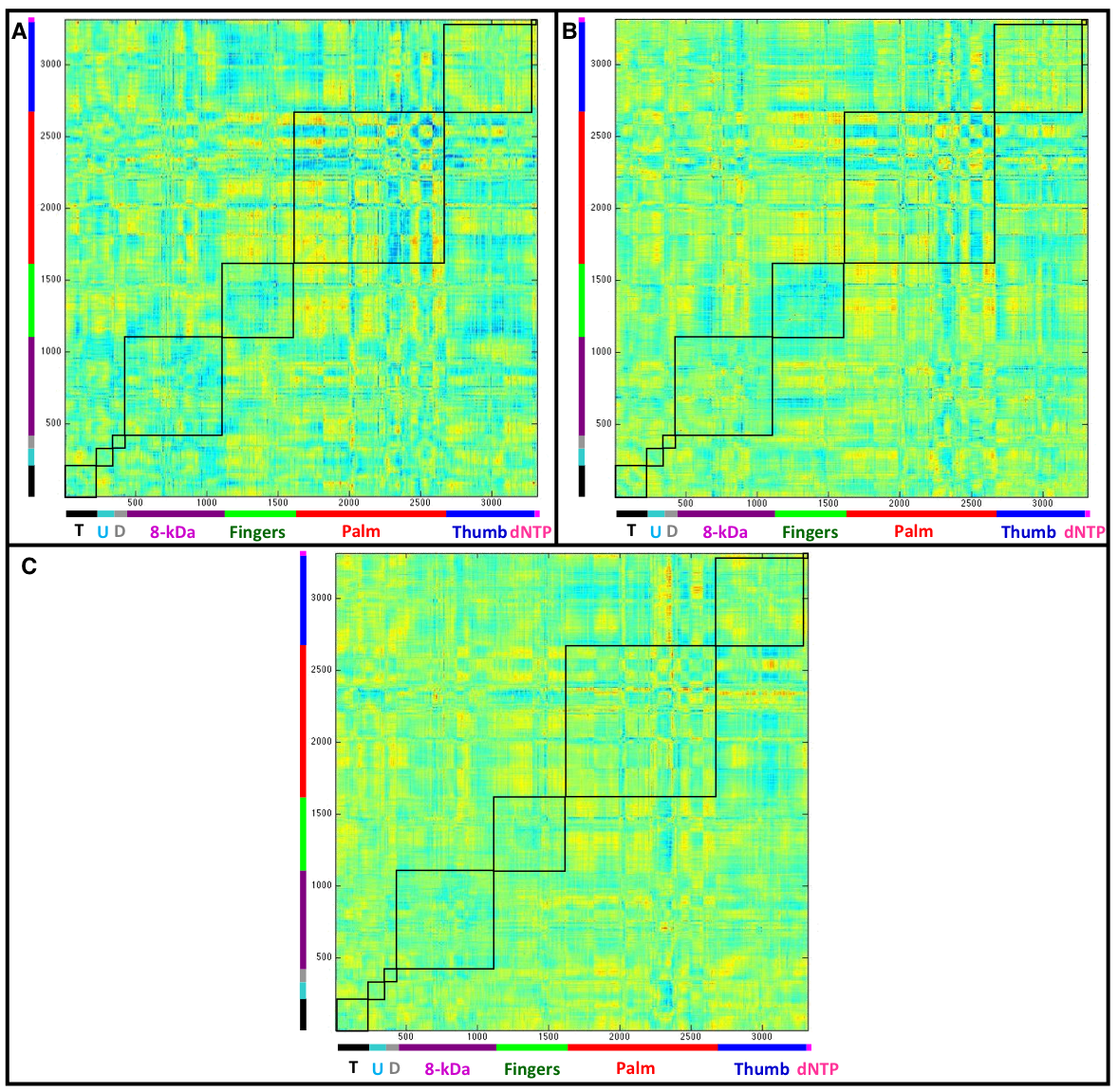

Supplement: Figure S8 — Difference covariance matrix for protein/DNA heavy atoms in non-cognate systems compared to A:dTTP system. (A) A:dATP; (B) A:dCTP; and (C) A:dGTP. Lighter color represents less difference compared to A:dTTP system. T, DNA template strand; U, DNA upstream primer strand; D, DNA downstream primer strand. Colors: black (template), cyan (upstream primer), silver (downstream primer), purple (8-kDa domain), green (fingers), red (palm), blue (thumb), and magenta (dNTP). (TIF) [file pcbi.1003074.s008.tif]

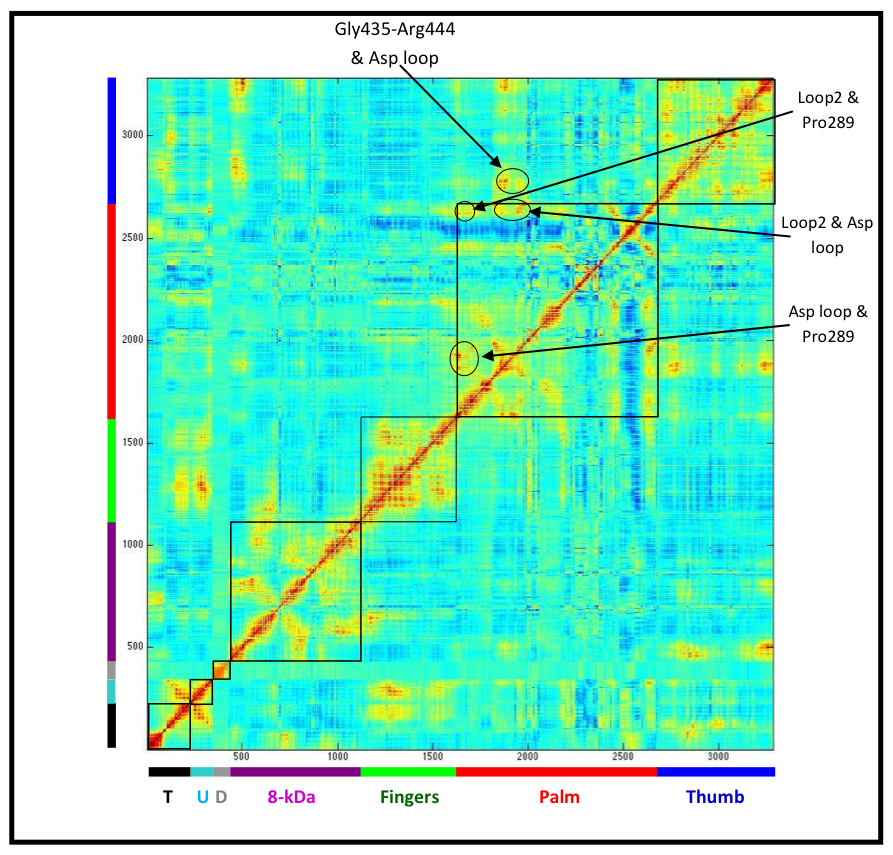

Supplement: Figure S9 — Covariance matrix for protein/DNA heavy atoms in nucleotide-absent system of pol μ. T, DNA template strand; U, DNA upstream primer strand; D, DNA downstream primer strand. Colors: black (template), cyan (upstream primer), silver (downstream primer), purple (8-kDa domain), green (fingers), red (palm), and blue (thumb). (TIF) [file pcbi.1003074.s009.tif]

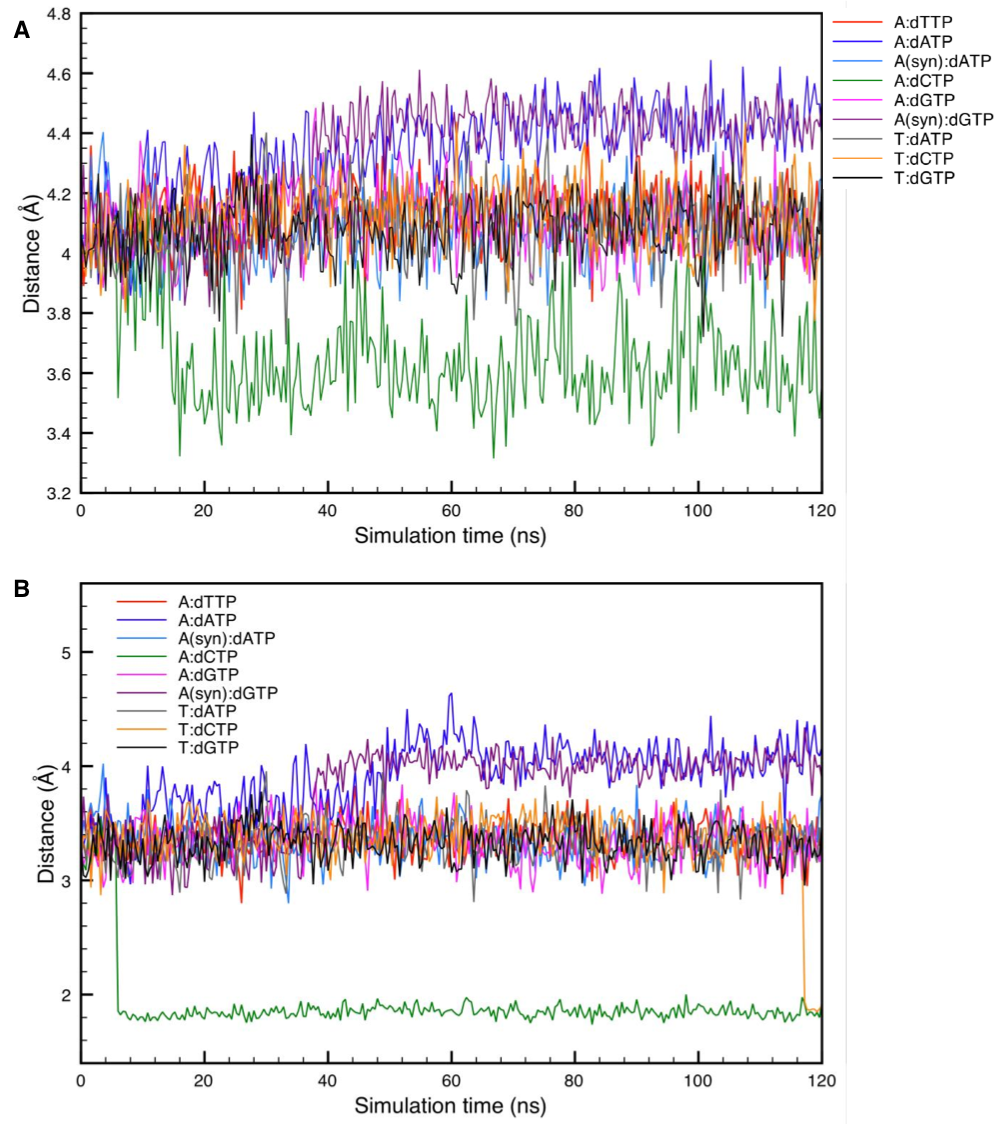

Supplement: Figure S10 — Plots of distance data of Mg2+. (A) Distance between Mg2+ (A) and Mg2+ (B) in all cognate and non-cognate systems; (B) Distance between Mg2+ (A) and dNTP:O1A in all cognate and non-cognate systems. (TIF) [file pcbi.1003074.s010.tif]
