# Supplementary material for: “Gate-keeper” Residues and Active-Site Rearrangements in DNA Polymerase μ Help Discriminate Non-cognate Nucleotides
Source: PLoS Comput Biol. 2013 May 23;9(5):e1003074. doi: 10.1371/journal.pcbi.1003074 (PMC3662701; doi:10.1371/journal.pcbi.1003074)
Supplement: Text S1 — Details on protonation states and correlated motion analysis. (PDF) [file pcbi.1003074.s011.pdf]

## Supporting Information

### Protonation States

We choose protonation states of the titratable side chains and phosphate groups in pol β based on individual pKa values at a solution pH of 7.0 as reported in Table S2. Because the three conserved Asp groups are well separated from each other and not closely interacting with the dCTP in the crystal structure, our choice of the protonation states based on pKa of the amino acid group and an overall pH of 7.0 is reasonable. It is also in accord with previous work [1-7]. We choose HID (His with hydrogen on the delta nitrogen) for all His groups. In prior work [6], we found that the choice of His tautomers for His329 in pol μ does not affect the active-site rearrangement and nucleotide-binding pocket motion, so this choice of His tautomers is expected to have have little impact on the results of this paper.

### Correlated Motion Analysis

The extent of correlated motions (positive or negative) for two molecular components can be quantified by calculating the covariance between the two units (e.g., atoms, residues, or subdomains) [8]. To investigate correlated motions among the DNA polymerase, DNA, and the incoming nucleotide, we calculated the normalized covariance, $C_{ij}$, for the displacement of all heavy atom pairs, I and j, as given by:

$$C_{ij} = \frac{\left\langle \Delta r_i \cdot \Delta r_j \right\rangle}{\left( \left\langle \Delta r_i^2 \right\rangle \left\langle \Delta r_j^2 \right\rangle \right)^{1/2}}$$

where $\Delta r_i$ is the displacement from the mean position of the i-th atom determined from all configurations in the trajectory segment being analyzed. $C_{ij}$ is normalized to be between –1 and 1. For completely correlated motions (i.e., when two atoms are moving in the same directions), $C_{ij} = 1$, and, for completely anti-correlated motions (i.e., when two atoms are moving in opposite directions), $C_{ij} = -1$ .

Covariance analysis is performed on the last 50 ns MD trajectories of A:dTTP, A:dATP, A:dCTP, and A:dGTP pol μ systems. For these covariance analyses, rotations and translations of the proteins occurring during the trajectories are removed by alignment to the initial protein conformation using a least-square-fit procedure for all protein heavy atoms.

## References

1. Sampoli Benitez BA, Arora K, Balistreri L, Schlick T (2008) Mismatched base-pair simulations for ASFV Pol X/DNA complexes help interpret frequent G*G misincorporation. Journal of Molecular Biology 384: 1086-1097.
2. Radhakrishnan R, Schlick T (2006) Correct and incorrect nucleotide incorporation pathways in DNA polymerase beta. Biochemical and Biophysical Research Communications 350: 521-529.
3. Arora K, Beard WA, Wilson SH, Schlick T (2005) Mismatch-induced conformational distortions in polymerase beta support an induced-fit mechanism for fidelity. Biochemistry 44: 13328-13341.
4. Radhakrishnan R, Schlick T (2005) Fidelity discrimination in DNA polymerase beta: differing closing profiles for a mismatched (G:A) versus matched (G:C) base pair. J Am Chem Soc 127: 13245-13252.
5. Li Y, Gridley CL, Jaeger J, Sweasy JB, Schlick T (2012) Unfavorable electrostatic and steric interactions in DNA polymerase beta E295K mutant interfere with the enzyme's pathway. Journal of the American Chemical Society 134: 9999-10010.
6. Li Y, Schlick T (2010) Modeling DNA polymerase mu motions: subtle transitions before chemistry. Biophysical Journal 99: 3463-3472.
7. Foley MC, Schlick T (2009) Relationship between conformational changes in pol lambda's active site upon binding incorrect nucleotides and mismatch incorporation rates. J Phys Chem B 113: 13035-13047.
8. Ichiye T, Karplus M (1991) Collective motions in proteins: a covariance analysis of atomic fluctuations in molecular dynamics and normal mode simulations. Proteins 11: 205-217.
