# Supplementary material for: “Gate-keeper” Residues and Active-Site Rearrangements in DNA Polymerase μ Help Discriminate Non-cognate Nucleotides
Source: PLoS Comput Biol. 2013 May 23;9(5):e1003074. doi: 10.1371/journal.pcbi.1003074 (PMC3662701; doi:10.1371/journal.pcbi.1003074)
Supplement: Table S1 — Distance between “gate-keeper” residues and the upstream primer in pol β, pol λ, and pol μ complexes. (PDF) [file pcbi.1003074.s012.pdf]

Table S1. Distance between "gate-keeper" residues and the upstream primer in pol β, pol λ, and pol μ complexes.

	Distance in dNTP-free system (Å)[a]	Distance in cognate system (Å)	Average (Å)
**Pol β**			
Arg258	5.0	7.2	6.1
Phe272	7.5	4.7	6.1
**Pol λ**			
Tyr505	6.6	4.9	5.8
Phe506	5.7	5.1	5.4
Arg517	9.5	8.4	9.0
**Pol μ**			
Trp436[b]	4.2	3.7	4.0
Gln440	7.6	8.7	8.2
Glu443	8.8	9.6	9.2
Arg444	9.4	8.9	9.2

[a] Distance is chosen from the flexible end of each "gate-keep" residue to the nearest heavy atom of the upstream primer. Data shown are average values from 20 ns MD simulation of corresponding systems.

[b] Trp436 only functions as "gate-keeper" residue in one non-cognate system (A:dGTP).
