# Supplementary material for: “Gate-keeper” Residues and Active-Site Rearrangements in DNA Polymerase μ Help Discriminate Non-cognate Nucleotides
Source: PLoS Comput Biol. 2013 May 23;9(5):e1003074. doi: 10.1371/journal.pcbi.1003074 (PMC3662701; doi:10.1371/journal.pcbi.1003074)
Supplement: Table S2 — Protonation states of titratable side chains and phosphate groups in pol μ. (PDF) [file pcbi.1003074.s013.pdf]

Table S2. Protonation states of titratable side chains and phosphate groups in pol μ.

Residue	Charge	pKa
Phosphate groups in DNA	–1	1.2
Asp	–1	3.9
Glu	–1	4.3
His	0	6.5
Lys	+1	10.8
Arg	+1	12.5
